# Artificial intelligence-based action recognition and skill assessment in robotic cardiac surgery simulation: a feasibility study

**DOI:** 10.1007/s11701-025-02563-3

**Published:** 2025-07-13

**Authors:** Gennady V. Atroshchenko, Lærke Riis Korup, Nasseh Hashemi, Lasse Riis Østergaard, Martin G. Tolsgaard, Sten Rasmussen

**Affiliations:** 1 Department of Cardiothoracic Surgery, Aalborg University Hospital, Hobrovej 18-22, 9000 Aalborg, Denmark; 2 ROCnord Robotic Center Aalborg, Aalborg University Hospital, Aalborg, Denmark; 3 Department of Clinical Medicine, Aalborg University, Aalborg, Denmark; 4 NordSim, Aalborg University Hospital, Aalborg, Denmark; 5 Department of Health Science and Technology, Aalborg University, Aalborg, Denmark; 6 Copenhagen Academy for Medical Education and Simulation (CAMES), Center for HR & Education, Copenhagen, Denmark; 7 Department of Obstetrics, Copenhagen University Hospital Rigshospitalet, Copenhagen, Denmark; 8 Department of Clinical Medicine, Faculty of Health and Medical Sciences, University of Copenhagen, Copenhagen, Denmark

**Keywords:** Robotic cardiac surgery, Deep learning, Convolutional neural network, Surgical action recognition, Automated skill assessment

## Abstract

To create a deep neural network capable of recognizing basic surgical actions and categorizing surgeons based on their skills using video data only. Nineteen surgeons with varying levels of robotic experience performed three wet lab tasks on a porcine model: robotic-assisted atrial closure, mitral stitches, and dissection of the thoracic artery. We used temporal labeling to mark two surgical actions: suturing and dissection. Each complete recording was annotated as either “novice” or “expert” based on the operator’s experience. The network architecture combined a Convolutional Neural Network for extracting spatial features with a Long Short-Term Memory layer to incorporate temporal information. A total of 435 recordings were analyzed. The fivefold cross-validation yielded a mean accuracy of 98% for the action recognition (AR) and 79% for the skill assessment (SA) network. The AR model achieved an accuracy of 93%, with average recall, precision, and F1-score all at 93%. The SA network had an accuracy of 56% and a predictive certainty of 95%. Gradient-weighted Class Activation Mapping revealed that the algorithm focused on the needle, suture, and instrument tips during suturing, and on the tissue during dissection. AR network demonstrated high accuracy and predictive certainty, even with a limited dataset. The SA network requires more data to become a valuable tool for performance evaluation. When combined, these deep learning models can serve as a foundation for AI-based automated post-procedural assessments in robotic cardiac surgery simulation. ClinicalTrials.gov (NCT05043064).

## Introduction

Since the first robotic heart surgery was performed in Paris in 1998 [1], the initial slow adoption of robotic technology in cardiac surgery has rapidly expanded, particularly in recent years [2]. Today, various cardiac operations can be performed with robotic assistance for selected patients. These procedures include coronary artery bypass grafting [3], resection of cardiac tumors [4], valve surgery [5, 6], fibrillation surgery [7], and even heart transplants [8].

Simulation plays a crucial role in modern robotic surgical training and typically includes dry lab, virtual reality, or wet lab training [9]. Consequently, there has been a growing demand to standardize robotic surgical training [10, 11] with a heightened focus on objectively assessing robotic skills [12–14]. To objectively assess robotic surgical performance, various evaluation tools have been developed, such as the Robotic Objective Structured Assessment of Technical Skills (R-OSATS) [15], the Global Evaluative Assessment of Robotic Skills (GEARS) [16] score, and the Time-based score (TBS) [17]. However, using these assessment tools requires assistance from an experienced robotic surgeon, which can be resource-demanding, time-consuming, and susceptible to interrater variability [18].

Artificial intelligence (AI) is an emerging and promising tool to guide assessment in robotic surgery [19]. Reports indicate that AI can provide more reliable assessments compared to traditional score evaluations and can reduce reliance on expert [20]. Recently, there has been increased research into robotic action recognition and performance assessment using machine learning (ML) and deep learning (DL) [18], and subfields of AI [21]. AI models capable of automatically identifying different stages of surgical performance in robotic-assisted surgery (RAS) from video recordings are the object of intense investigation [22, 23]. However, current data on artificial intelligence in robotic surgery indicate that automated assessment tools are not well validated and that AI-based assessments are still in a conceptual stage [19]. The effectiveness of AI models is highly dependent on the quality, quantity, and variety of their datasets [24]. Given the increasing demand for objective assessment in robotic cardiac surgery [10], robust ML algorithms trained on high-quality data are essential. To the best of our knowledge, there are currently no data available on the use of AI for video-based assessment of surgical actions and performance in robotic cardiac surgery.

In this feasibility study, we introduce a method that utilizes video-based data from the robotic cardiac surgery wet lab simulation training to create a deep neural network capable of recognizing basic surgical actions and categorizing surgeons based on their performance level.

## Materials and methods

This study used video data from the robotic cardiac surgery wet lab simulation trial (ClinicalTrials.gov: NCT05043064) conducted at the ROCnord robotic center of Aalborg University Hospital in Aalborg, Denmark. The detailed design of this trial has been reported previously [25, 26].

**Fig. 1:**
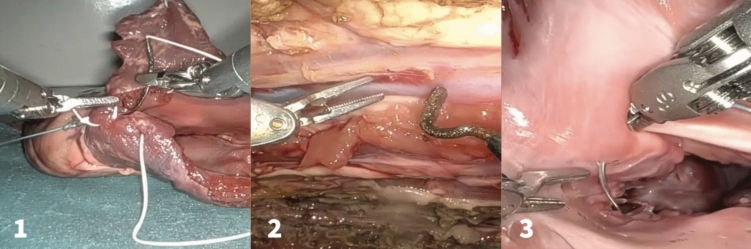
Wet lab simulation tasks (adapted from Atroshchenko et al. [26]). 1 Robotic-assisted closure of the left atrium. 2 Robotic-assisted dissection of the internal thoracic artery. 3 Robotic-assisted placement of the mitral annular stitches

In summary, four experienced robotic cardiac surgeons and 15 surgeons without robotic experience performed three wet lab tasks in the porcine model: robotic-assisted closure of the left atrium, mitral stitches placement, and internal thoracic artery dissection (Fig. 1) [26]. Each task was video recorded and evaluated using TBS and GEARS scores. Participants trained until they achieved mastery based on the TBS.

### Data capture and preparation

For data extraction and preparation, we used the method described by Hashemi et al. [27]. Video footage was recorded using OBS Studio (Open Broadcaster Software Studio) from the da Vinci surgical console through Ozvavzk’s Video Capture Card. The recording set was 30 frames per second (FPS) with a resolution of 1920 × 1080 pixels, capturing footage from either the right or left ocular, depending on the surgeon’s hand dominance. All irrelevant sequences, such as the changing or cleaning robotic instruments, have been removed.

We utilized temporal labeling to mark two basic surgical actions: suturing and dissection, employing the Behavioral Observation Research Interactive Software (BORIS, v. 7.13.8). We categorized both single stitches (for each mitral annular stitch) and running sutures (for atrial closure) under the primary label of “suturing.”

A single stitch annotation began when the needle tip entered the tissue and concluded when the base of the needle was pulled out of the tissue. Handling the needle outside of the tissue was labeled separately and was not included in the single stitch annotation. If the surgeon started to push the needle through the tissue but then chose to extract it, the movement was still labeled as a stitch, ending when the needle tip was pulled out again.

For a running suture, we used a specific annotation code that begins at the start of the first stitch and ends when the last stitch is pulled from the tissue. The running suture was further divided into individual stitches, following the same principle as with single sutures. Only the time the needle remained in the tissue was labeled as a single suture; the remainder of the time was categorized as handling the needle. Dissection was annotated during the period when diathermy was applied to the tissue. Blunt dissections were labeled from the moment the instrument made contact with the tissue until the movement stopped. Movements of the diathermy that did not involve dissections were labeled as “instrument handling”.

To evaluate SA, we focused on recordings of the mitral stitches and the atrial closure tasks from the first attempt only. These tasks were selected, because they demonstrated significant differences in TBS and GEARS scores between the novices and experts in our previous trial [25]. This approach ensured that only recordings with clearly differentiated performance levels were used for AI analysis. Each complete recording was labeled as either “novice” or “expert” based on the operator’s experience.

All video footage and labels have been anonymized and are available as open-source under the project name “Robotic cardiac surgery WetLab” at www.synapse.org.

### Dataset pre-processing

For pre-processing and deep learning, we followed the method outlined by Hashemi et al. [28]. For action recognition, we used a frame rate of 1 FPS and organized the video footage into sequences of five consecutive frames. To assess performance levels, we used sequences of 10 s. Both processes were implemented using Python 3.9. Additionally, the footage was cropped and resized to 256 × 256 pixels to decrease computational load.

The complete dataset was balanced and divided into three sets: training, validation, and test sets. Approximately 80% of the data were used for training, while the remaining 20% was split equally between the validation and test sets. The splitting and balancing were performed automatically using a Python script. For SA, the dataset was manually balanced to include both novices and experts across all three sets. We ensured that each participant’s videos were used exclusively in one specific dataset, meaning that no participant appeared in more than one dataset at the same time.

### The deep neural network

**Fig. 2:**
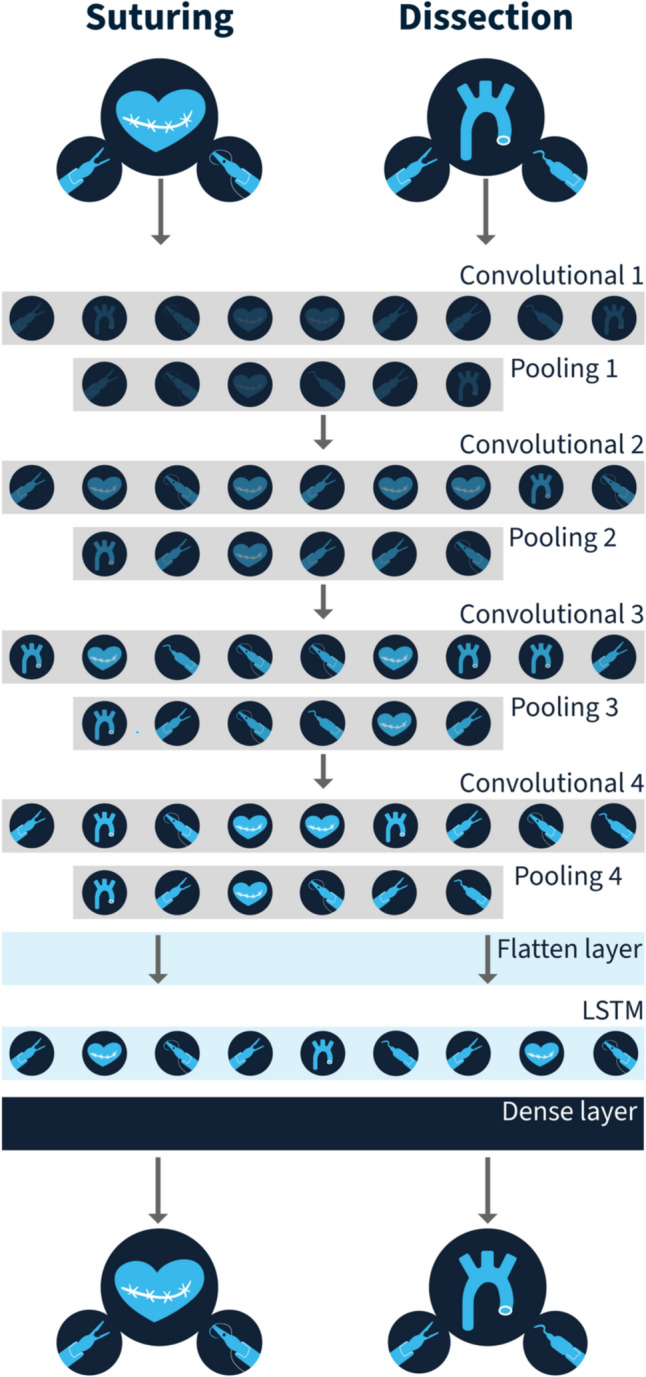
Architecture of the deep neural network. *LSTM* Long Short-Term Memory layer

The network architecture (Fig. 2) combined a Convolutional Neural Network (CNN) for extracting spatial features with a Long Short-Term Memory (LSTM) layer to incorporate temporal information. The initial input layer was followed by four convolutional layers, each accompanied by pooling layers. After the convolutional processing, the output was flattened and fed into the LSTM. Finally, the output was processed through a dense layer that provided two probabilities: either suturing or dissection, or expert or novice.

We developed two versions of the network: one for surgical recognition and another for assessing performance levels. To mitigate overfitting and address the limited data, we also applied a dropout rate of 0.20, batch normalization, and an L2 regularization rate of 0.01.

We used a batch size of eight sequences for training, and a maximum of 50 epochs were conducted, utilizing early stopping to prevent overfitting.

### Model evaluation

We employed K-fold cross-validation with fivefold on the combined training and validation sets to identify the optimal hyperparameters for our model. These hyperparameters were then used to train a new AI model from scratch, which was evaluated on an unseen test set.

To assess our models, we generated confusion matrices and calculated various metrics, including accuracy, recall, precision, F1-score, and true-positive and false-positive rates. To enhance interpretability, we calculated the predictive certainty of the network by making predictions and obtaining probabilities for each class (dissection vs. suturing and novice vs. expert). We then recorded the highest probability for each prediction and compared it to the corresponding ground truth labels. The probability distribution represents the certainty behind classifying a sequence into a specific class. Additionally, we applied Gradient-weighted Class Activation Mapping (Grad-CAM) to visualize the most influential regions in the image sequences analyzed by the algorithm.

## Results

A total of 435 recordings, amounting to 35 h, 18 min, and 56 s, were analyzed and temporally annotated for surgical action recognition. These recordings were divided into two groups: suturing and dissection. Among them, 39 procedural videos in the suturing group were labeled as “expert,” while 30 videos were labeled as “novice.”

Results from K-fold cross-validation are presented in Table 1, yielding a mean accuracy of 98% for action recognition and 79% for SA.

**Table 1:** Accuracy results of K-fold cross-validation for action recognition and skill assessment

	Action recognition (%)	Skill assessment (%)
Fold 1	97.1	55.3
Fold 2	99.8	99.9
Fold 3	100	70.5
Fold 4	96.9	83.7
Fold 5	98.7	85.3
Mean accuracy (± SD)	98.5 (± 1.5)	78.9 (± 16.8)

*SD* standard deviation

The deep learning network achieved the lowest average validation loss for primary action classification in the 5th epoch and for SA in the 24th epoch. The final models from these epochs were saved for testing.

The action recognition model achieved an accuracy of 93%, while the skill assessment deep learning network had an accuracy of 56%. The evaluation metrics of the deep learning network for predicting the test set are shown in Table 2. The predictive certainty for AR was 99%, with a maximum of 100% and a minimum of 62%. For SA, the predictive certainty was 95%, with a maximum of 99.9% and a minimum of 57%.

**Table 2:** Evaluation metrics of the deep learning network to predict the test set

	Recall	Precision	F1-score
Action recognition			
Suturing	0.86	0.99	0.92
Dissection	0.91	0.89	0.94
Weighted average	0.93	0.93	0.93
Skill assessment			
Expert	0.1	1.00	0.19
Novice	1.00	0.53	0.69
Weighted average	0.56	0.76	0.44

**Fig. 3:**
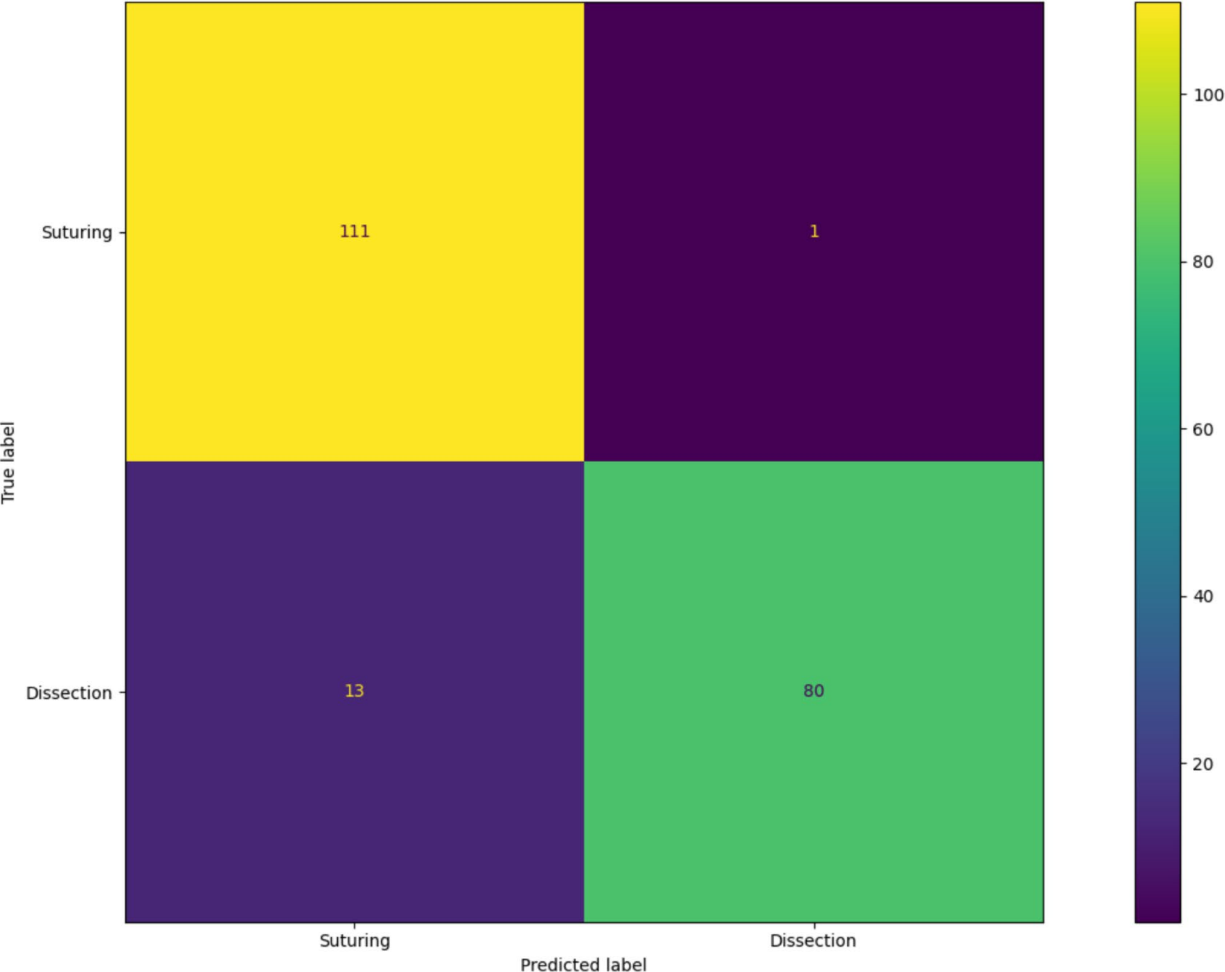
The confusion matrix for the action recognition network

The confusion matrix in Fig. 3 illustrates the gesture recognition DL network’s accuracy. The model demonstrated high accuracy, showing a low number of false-positive and false-negative predictions.

**Fig. 4:**
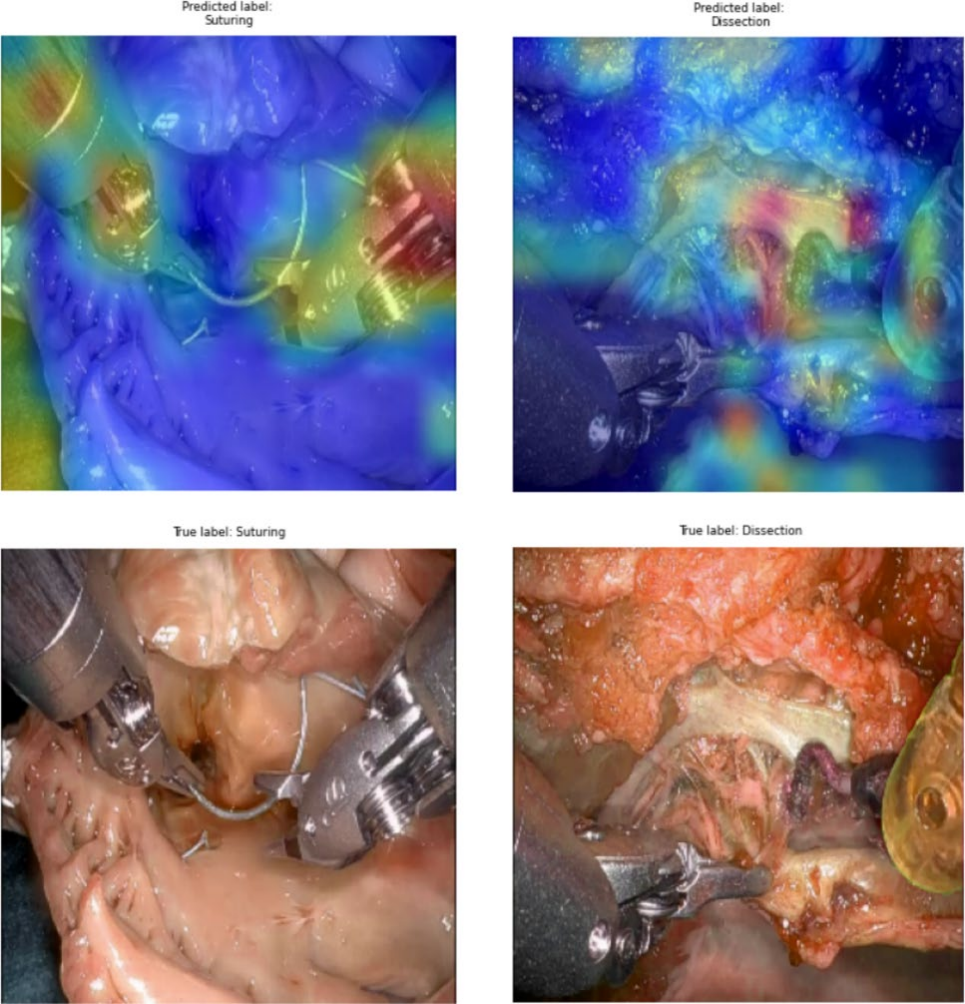
Gradient-weighted Class Activation Mapping for the action recognition network (Grad-CAM). Grad-CAM filter shows that the model focuses on the needle, suture, and instrument tips during suturing (top left image) and on the tissue during dissection (top right image). The lower row displays the corresponding video frames analyzed by the model

Gradient-weighted Class Activation Mapping for the action recognition deep learning network (Fig. 4) revealed that the algorithm focused on the needle, suture, and instrument tips during suturing, and on the tissue during dissection.

## Discussion

In this feasibility study, we trained two deep learning networks using video data from robotic cardiac surgery wet lab simulation training, employing a novel AI algorithm. The AR network demonstrated reliable performance in accurately classifying surgical actions, even with a limited dataset. The SA network requires more data to become a useful model for performance evaluation. When combined, these deep learning models can facilitate automated post-procedural assessments in robotic cardiac surgery simulation. We are the first to present a deep learning-based approach for classifying surgical actions and skills in robotic cardiac surgery using only video data.

The overall accuracy of gesture recognition methods is reported to be approximately 80% in mixed datasets that use kinematic signals and video data [29]. Deep neural networks capable of automatically capturing complex temporal dependencies in surgical motion appear to perform better than traditional computer vision methods [30, 31]. State-of-the-art models that use video data for robotic action recognition, such as 3D ConvNet [22] and the unified Surgical AI System (SAIS) [23], have achieved high skill classification accuracies ranging from 95 to 100%. However, the 3D ConvNet model was trained and tested on the JIGSAWS [29] dataset collected from 8 right-handed subjects performing 39 suturing, 36 knot-tying, and 20 needle-passing in a dry lab. The SAIS dataset is not publicly available, and the model was specifically trained, validated, and tested on urological robotic procedures. Our AR network, trained on cardiac surgical tasks conducted by 19 subjects with varying hand dominance, demonstrated high performance based on comprehensive evaluation metrics, including recall, precision, F1 score, and confusion matrix, comparable to the aforementioned models. The K-fold cross-validation, which provides a more generalized estimate of how the model would perform on unseen data, revealed strong performance (a mean accuracy of 98%), demonstrating the feasibility of DL-based recognition of surgical actions. Unlike models trained on the JIGSAWS dataset, which focuses on generic dry lab tasks, our AR model trained on the procedure-specific cardiac surgical tasks, such as the internal thoracic artery dissection, making the network applicable to robotic cardiac surgery.

However, the skill assessment DL network did not achieve the desired characteristics, even after additional fine-tuning and the addition of an extra dense layer, which had proven effective in another study using the same algorithm [28]. One possible explanation for this underperformance may be the limited number of experienced robotic surgeons—only four, compared to 15 novices. This disparity may lead to skewness in the variation that the skill assessment model attempts to learn. This is evidenced by the higher standard deviation of cross-validation for skill assessment performance compared to that of the AR network across the folds (16.8 versus 1.5, respectively). This small, unbalanced dataset likely limits the SA model’s generalizability. A potential solution would be to record more exercises performed by expert robotic cardiac surgeons to gather additional data and retrain the SA network. However, several challenges complicate this approach. These include the limited number of expert surgeons available globally, their unavailability for training sessions, the absence of a standardized robotic cardiac training curriculum [25, 32], high costs, restricted access to animal models, and the availability of robotic platforms for training [26]. These factors make implementing this solution quite difficult. Prior studies in the field of surgical data science have produced varying dataset sizes. For example, Kiyasseh et al. [23] analyzed 78 robot-assisted radical prostatectomies performed by 19 surgeons. In contrast, large-scale image recognition datasets, such as the ImageNet [33], contain over 1 million labeled images across 1,000 object categories. Developing robust, scalable models will require large datasets, ideally drawn from multiple institutions, and procedure types. Key considerations include ensuring annotation consistency, addressing patient privacy, and developing shared platforms to enable widespread data sharing and model training [34, 35]. Another challenge in collecting data for SA is that labeling based solely on prior experience is a weak proxy for actual skill. As a result, out of a total of 435 videos used for AR, only 69 were selected for SA. These recordings were chosen because their scores on TBS and GEARS demonstrated sufficient validity evidence [25]. This approach ensured that only recordings with clearly differentiated performance levels were used for AI analysis. We did not include the ITA dissection for SA, as both TBS and GEARS metrics appeared to be unreliable in distinguishing between experienced and inexperienced robotic cardiac surgeons. Future research should focus on defining what constitutes an “expert” robotic cardiac surgeon whose performance is deemed “acceptable” for training AI models, as well as determining which authorities will make these assessments.

While RAS is performed by human surgeons, the development of AI technologies for surgical applications is usually carried out by biomedical engineers or computer scientists [31]. If the models are not presented in a way that surgeons as non-specialists can easily understand, they may struggle with the scientific terminology, leading to a reluctance to use these tools [24]. The Grad-CAM is used to highlight the areas the network focuses on during training (Fig. 4). It aims to enhance the interpretability of deep learning algorithms, as a lack of clear explanations for their functioning can hinder their adoption in healthcare [36, 37]. Another significant component in our network is LSTM layer, which enables the model to recognize patterns over time rather than just isolated frames [28]. When considering the temporal aspect of surgical videos, the model can analyze smaller, sequential segments of the procedure rather than relying on single-frame analysis. This allows for more granular assessments of surgical performance, enabling the evaluation of each moment during surgery. Recent studies have demonstrated the effectiveness of this temporal modeling, using LSTM method among others [23, 28]. As a result, visualizations like procedural plots or surgical skill profiles have emerged, illustrating both specific parts and entire procedures [23]. Importantly, this method moves us closer to real-time skill assessment, where the temporal aspect is needed for continuous feedback during an ongoing surgical procedure. By moving beyond static frame analysis and embracing the dynamic nature of surgical performance, LSTM-based models provide a more nuanced and clinically relevant understanding of technical skill.

In our study, shorter five-frame sequences were chosen for action recognition, in line with previous research indicating that surgical gestures can be reliably classified within 2–12 s of motion [31, 38]. These shorter clips are often sufficient for detecting specific motor patterns or transitions, while also reducing computational demands. In contrast, skill assessment is a more nuanced and temporally extended task. Prior studies have used sequences of 10 s, showing that skill levels can be differentiated within this timeframe [22, 39]. We, therefore, selected 10-s clips to strike a balance between capturing meaningful skill-related dynamics and maintaining manageable sequence lengths for training.

Although wet lab training is an essential component of robotic cardiac surgical education [40], and since there are currently no virtual reality simulators that encompass cardiac surgeries [41], we selected a cardiac wet lab simulation model [25] for our study. We recorded surgical performance data from the da Vinci Xi (Intuitive Surgical) robotic console. However, the method [27] we used for video data extraction and preparation can easily be applied to any other robotic system. This makes the data collection independent of the specific surgical system, especially since more surgical robots have emerged recently [42].

Currently, there is no standardized curriculum for robotic cardiac surgery training in either Europe or North America [10, 32, 43]. However, there is widespread agreement that wet lab simulation is a crucial component of this training [40]. Training needs assessment. The previously discussed challenge of the availability of experienced robotic cardiac surgeons for assessment drives the search for robust and reliable alternatives. While AI-based models have been rigorously investigated in other surgical specialties to address this issue, the optimal solution has yet to be determined [19]. An ideal AI-based system could automatically identify different stages of surgical performance from videos by analyzing surgical activities, gestures, and skill levels, providing real-time feedback to trainees and reducing their reliance on expert surgeons [20, 23]. Our feasibility study introduces a new DL model trained on robotic cardiac surgery simulation data, which could potentially be used for post-procedure feedback analysis. While the AR network demonstrated its feasibility, further testing is necessary to determine whether these findings are generalizable to unseen videos from different surgeons. A more comprehensive effort, including stronger annotation criteria, richer task diversity, and more rigorous evaluation would be necessary to enhance the model performance. Future research would benefit from a multicenter approach to gathering more data, addressing the challenge of having a limited number of experienced robotic cardiac surgeons at a single center.

## Strengths and limitations

One of the strengths of this study is that our approach is likely applicable to other RAS systems. The algorithm we used to train the DL network has already proven its feasibility for video-based AR and SA in general surgery [28], demonstrating the generalizability of the model. Additionally, we have explained how the data were split into training, validation, and test sets, ensuring that there was no leakage between these sets and that no participant appeared in more than one dataset at the same time.

However, there are some limitations to note. Only one human rater annotated all the videos, which introduces a potential source of bias. For our action recognition, we only decoded two surgical actions—suturing and dissection—thus limiting the network’s overall utility. Moreover, the small dataset used for the skill assessment model resulted in low accuracy during testing. Due to the limited size of our dataset and computational resources, we opted for a commonly used architecture and tuned hyperparameters based on performance on the validation set, rather than using exhaustive search. While we conducted basic analyses, including confusion matrices, we acknowledge that a more detailed error analysis—such as calibration curves or entropy-based confidence evaluations—would enhance the interpretation of these results.

## Conclusions

In this feasibility study, we developed deep learning networks for surgical action recognition and skill assessment using only video data from robotic cardiac surgery wet lab simulation. The action recognition network demonstrated high accuracy and predictive certainty, even with a limited dataset. The skill assessment network requires more data to become a valuable tool for performance evaluation. When combined, these deep learning models may serve as a foundation for AI-based automated post-procedural assessments in robotic cardiac surgery simulation.

## Data Availability

All video footage and labels have been anonymized and are available as open-source under the project name “Robotic cardiac surgery WetLab” at www.synapse.org.

## Abbreviations

AI: Artificial intelligence
AR: Action recognition
CNN: Convolutional neural network
DL: Deep learning
FPS: Frames per second
GEARS: Global evaluative assessment of robotic skills
Grad-CAM: Gradient-weighted class activation mapping
ITA: Internal thoracic artery
LSTM: Long short-term memory
ML: Machine learning
RAS: Robotic-assisted surgery
SA: Skill assessment
SAIS: The surgical artificial intelligence system
TBS: Time-based score
